# The Use of Brain Stimulation in Dysphagia Management

**DOI:** 10.1007/s00455-017-9789-z

**Published:** 2017-03-28

**Authors:** Andre Simons, Shaheen Hamdy

**Affiliations:** 1 Manchester Medical School, University of Manchester, Manchester, UK; 2 GI Sciences, Division of Diabetes, Endocrinology and Gastroenterology, School of Medical Sciences, Faculty of Biology, Medicine and Health, Salford Royal Hospital (Part of the Manchester Academic Health Sciences Centre (MAHSC)), University of Manchester, Clinical Sciences Building, Eccles Old Road, Salford, M6 8HD UK

**Keywords:** Brain, Deglutition, Deglutition disorders, Dysphagia, Magnetic, Neurogenic, Neurostimulation, Rehabilitation

## Abstract

Dysphagia is common sequela of brain injury with as many as 50% of patients suffering from dysphagia following stroke. Currently, the majority of guidelines for clinical practice in the management of dysphagia focus on the prevention of complications while any natural recovery takes place. Recently, however, non-invasive brain stimulation (NIBS) techniques like transcranial magnetic stimulation (TMS) and transcranial direct current stimulation (tDCS) have started to attract attention and are applied to investigate both the physiology of swallowing and influences on dysphagia. TMS allows for painless stimulation of the brain through an intact skull—an effect which would normally be impossible with electrical currents due to the high resistance of the skull. By comparison, tDCS involves passing a small electric current (usually under 2 mA) produced by a current generator over the scalp and cranium external to the brain. Initial studies used these techniques to better understand the physiological mechanisms of swallowing in healthy subjects. More recently, a number of studies have investigated the efficacy of these techniques in the management of neurogenic dysphagia with mixed results. Controversy still exists as to which site, strength and duration of stimulation yields the greatest improvement in dysphagia. And while multiple studies have suggested promising effects of NIBS, more randomised control trials with larger sample sizes are needed to investigate the short- and long-term effects of NIBS in neurogenic dysphagia.

## Introduction

For the majority of people, swallowing is an effortless motor activity which is performed hundreds of times per day. However, despite its apparent simplicity it is considered to be one of the most complicated neuromuscular activities requiring the use of 26 pairs of muscles, five cranial nerves and several central nervous system processing levels [1]. As a result of its complexity, this physiological process is very susceptible to impairment if there is structural or neurogenic damage resulting in dysphagia.

Difficulty swallowing is common sequela of brain injury with as many as 50% of patients suffering from dysphagia following stroke [2]. Currently, the majority of guidelines for clinical practice in management of dysphagia focus on the prevention of complications while any natural recovery processes take place. Examples of this include compensatory manoeuvres like the chin tuck, supraglottic swallow and effortful swallow, and bolus modification to adjust the temperature, acidity, volume and viscosity of the bolus [3]. However, given the neural repair mechanisms that are likely to be involved in the recovery process, there has been increased interest in the role of neuromodulation to treat swallowing problems. Indeed, most recently, non-invasive brain stimulation techniques have started to attract attention and have begun to be used to investigate both the physiology of swallowing and influences on dysphagia. This review aims to give a historical perspective on non-invasive brain stimulation and its uses in the management of dysphagia.

### Transcranial Magnetic Stimulation (TMS)

**Fig. 1:**
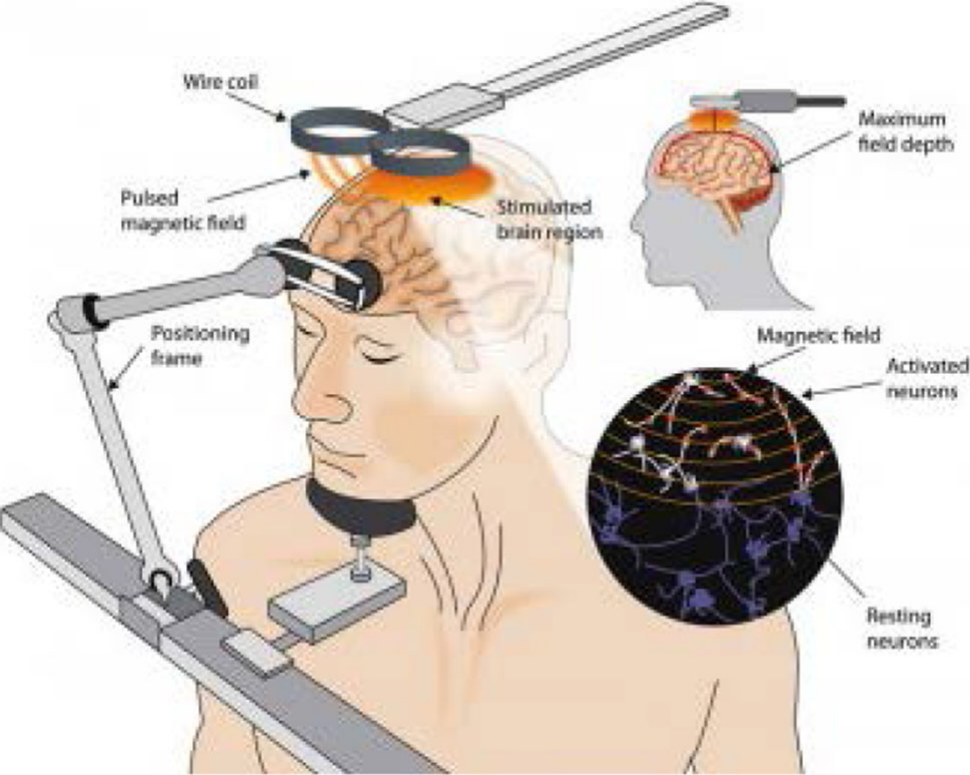
Illustration of the technique of transcranial magnetic stimulation (taken from https://thebrainstimulator.net/brain-stimulation-comparison/)

It has long been known that nerves and muscles can be stimulated with externally applied electrical currents, and since the work of Polsen et al. and Barker et al. in 1982 and 1985, respectively, magnetic stimulation techniques have been developed and refined [4]. TMS allows for painless stimulation of the brain through an intact skull—an effect which would normally be impossible with electrical currents due to the high resistance of the skull [5]. Indeed, much of what we understand today about TMS harks back to the work of Michael Faraday who demonstrated that a rapidly changing magnetic field can induce current flow: known as Faraday’s 3rd law of electromagnetism [6]. Operationally, TMS is performed using a pulse generator which passes a very large (>5 kA) but very brief (<1 ms) current through a coil placed directly above the subject’s head [7]. The current flowing through the coil generates a magnetic field which is similar in size to that of a magnetic resonance imaging scanner [5], and this in turn generates small eddy currents in the brain tissue which travel perpendicular to the direction of the magnetic field. These small currents are sufficient to cause depolarisation of axons in the cortex and subcortical white matter (Fig. 1). Stimulation with TMS is not particularly precise due to the diverging magnetic field; however, stimulation can be focused more by using two circular coils to form a figure-of-eight coil. The magnetic field is summed up at the point of intersection of the two coils [7].

One of the first studies to utilise TMS related to swallowing was a physiological experiment by Valdez et al. in 1993. Single-pulse TMS was delivered to three healthy dogs at intervals ranging from 15 s to 3 min. The researchers found that the upper oesophagus sphincter twitched when the magnetic stimulation was delivered and the amplitude of the twitch corresponded with increasing magnetic stimulation intensity. Valdez et al. concluded from their study that magnetic stimulation of the cerebral cortex can induce swallowing activity and that further studies could investigate the effects of TMS in humans [8].

Aziz et al. followed on from the work of Valdez in 1994 and 1995 by studying the oesophagus electromyographic (EMG) responses to magnetic stimulation of the human cortex and extracranial vagus nerve. In these studies, Aziz et al. used single-pulse TMS in a number of experiments stimulating the human cortex and this resulted in early and late EMG responses. It was found that higher intensity stimulation did not change the late response but increased the duration and amplitude of the early response. They also studied the effect of stimulation whilst subjects were performing the Valsalva manoeuvre and found that the early response was greater under these conditions while the late response remained unchanged [9, 10].

Hamdy et al. followed these studies up, exploring the physiological characteristics of the pathways from the cortex to oesophagus, pharyngeal and oral musculature in 1996 with TMS and recording EMG responses in 20 healthy individuals. Their study showed that the muscle groups involved in swallowing are somatotopically represented around the precentral which suggests that the motor cortex plays a larger role in swallowing than previously thought. Their study also highlighted that motor control is represented asymmetrically between the two hemispheres and data from two stroke patients in the same study also provided insight into this. The dysphagic stroke patient’s intact hemisphere had a significantly smaller area of pharyngeal representation than that of the non-dysphagic stroke patient. Hamdy et al. postulated that the presence of a dominant hemisphere for swallowing was independent of handedness, and if damaged would result in the patient suffering with dysphagia [11].

Repetitive TMS (rTMS) is a variation of TMS which involves the use of multiple pulses of equal intensity at a specific frequency. rTMS has been shown to cause changes in cortical excitability not only during stimulation but also for several minutes afterwards [12–14]. Studies, as with those described above, had shown that direct corticobulbar projections from the motor cortex to the swallowing musculature exist and that these projections are bilateral—albeit with a non-dominant side and a dominant side which has greater control over swallowing [15–19]. In progressing these studies further, Gow et al. in 2004 found that stimulation of the pharyngeal motor cortex with rTMS at 5 Hz caused increased excitability of the corticobulbar pharyngeal projections lasting over 60 min [18]. Later, in Mistry et al’s studies, it was shown that stimulation with high intensity 1 Hz rTMS had an inhibitory effect on the pharyngeal motor cortex for up to 45 min [20]. These temporary “virtual lesions” could then be used to test the efficacy of these neurostimulation techniques before they are trialled in affected patients.

In 2009, Jefferson et al. utilised the technique of creating a virtual lesion using 1 Hz rTMS and then tried to reverse the effect by a separate intervention. 23 healthy subjects were subjected to 1 Hz rTMS to create a virtual lesion in the pharyngeal motor cortex in one cerebral hemisphere. 5 Hz rTMS targeted over the unaffected cerebral hemisphere reversed the effect of the virtual lesion and the effects lasted for up to 50 min [21]. This work has since led to a series of more recent studies using excitatory rTMS to treat patients with dysphagia.

By contrast, a study in 2009 by Verin and Leroi used an alternative method of rTMS stimulation to improve swallowing function. In this study, experimenters used inhibitory 1 Hz rTMS to suppress the healthy hemisphere in patients who had suffered stroke. They observed an improvement in swallowing function on videofluoroscopy as well as improvement in reaction time tasks. However, this was a very small study and there was no control group [22].

Khedr et al. carried out two double-blinded randomised trials with rTMS in subacute stroke patients. Their 2009 study randomly allocated 26 patients with post-stroke dysphagia to receive either real rTMS (14 patients) or a sham procedure (12 patients). Patients received 300 pulses of 3 Hz rTMS at 120% of resting motor threshold intensity over the affected hemisphere for 5 days consecutively. Severity of dysphagia was assessed before the first and after the last session of rTMS, 1 month after the intervention and 2 months after the intervention. The study found that active rTMS improved symptoms of dysphagia compared with sham and improvements were maintained up to 2 months afterwards [23].

Khedr et al’s 2010 study included 22 brainstem stroke patients of which 11 were randomly assigned to receive active rTMS or sham stimulation of the oesophagus motor cortex. Their experimental procedure was largely the same as in their previous study except that the intensity of the rTMS was 130% of resting motor threshold and both hemispheres were stimulated. The findings of this study were consistent with the first [24].

Another study by Park et al. in 2013 investigated the effect of 5 Hz rTMS in patients with post-stroke dysphagia. This randomised controlled trial involved 18 patients all with unilateral hemispheric stroke and oropharyngeal dysphagia lasting over one month. Participants were divided into two groups randomly—an experimental group and a control group. The experimental group were subject to 5 Hz rTMS for 10 min per day for two weeks while the control group received sham rTMS for the same duration. The experimental group had clinical improvement in symptoms that lasted over 2 weeks after the trial. The researchers suggested that stimulation of the unaffected hemisphere facilitated the swallowing process by enhancing bulbar motor neuron stimulation to the pharynx [25].

Park et al. from a separate research group followed on from this work by comparing the effects of bilateral and unilateral rTMS. In their 2016 study, they randomly assigned 35 stroke patients with dysphagia to three intervention groups: bilateral stimulation group, unilateral stimulation group and sham stimulation group. The bilateral stimulation group received 500 pulses or 10 Hz rTMS daily for 2 weeks over both the ipsilesional and contralesional motor cortices projecting to the mylohyoid muscles. The unilateral stimulation group received 500 pulses of 10 Hz rTMS to the ipsilesional motor cortex and sham stimulation to the contralesional motor cortex. The sham group received bilateral sham stimulation to the motor cortices. Patients were assessed before the intervention, after the intervention and 3 weeks after the intervention using the dysphagia outcome and severity scale (DOSS), clinical dysphagia scale (CDS), videofluoroscopic dysphagia scale (VDS) and penetration aspiration scale (PAS). Patients receiving bilateral rTMS had the greatest improvements in swallowing function which led the authors to suggest that bilateral stimulation of the motor cortices projecting to the mylohyoid muscles is an effective dysphagia therapy. The decision to use 10 Hz stimulation as opposed to the more commonly used 5 Hz stimulation, and the decision to compare bilateral stimulation with ipsilesional excitatory stimulation as opposed to the more proven contralesional excitatory stimulation was somewhat unusual [26].

A problem with these studies is that, while promising, they are all of a small size and therefore subject to type 1 errors, making it difficult to establish whether the improvement in swallowing is directly related to the intervention or chance. Larger randomised controlled studies using rTMS in dysphagia will hopefully help to answer these questions in the future.

### Transcranial Direct Current Stimulation

The idea of using direct current to stimulate the body has been in existence for over 100 years, with Luigi Galvani’s experiments on frogs leading to the foundation of the study of electrophysiology [27]. Galvani’s nephew Giovanni Aldini pioneered the use of electrical stimulation in humans in 1801 when he applied electricity to an executed criminal’s head [28]. Direct current stimulation was used by D. J Albert in his experiments on cortical excitability in 1966. In the two papers he authored in 1966, Albert showed that anodal and cathodal electrical stimulation of a rat’s medial cortex could either speed up memory consolidation or reduce memory retention [29]. The methodology has since been refined and adopted for clinical use over the last two decades.

**Fig. 2:**
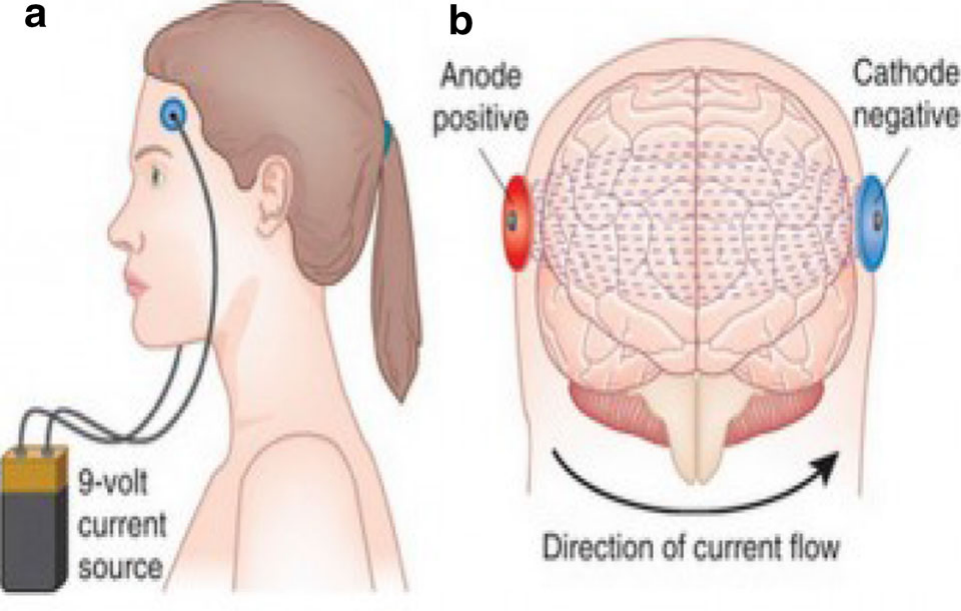
Illustration of tDCS (taken from https://thebrainstimulator.net/brain-stimulation-comparison/)

In contrast to TMS (see Table 1) tDCS involves passing a small electric current (usually under 2 mA) produced by a current generator over the scalp and cranium external to the brain (Fig. 2). The current is delivered through two conductive rubber electrodes covered in synthetic sponges soaked in saline [30]. These electrodes have a large surface area of around 20–35 cm^2^ which makes it difficult to focus the stimulation. The large surface area is necessary to keep the current density low, thus the subject only perceives a tingling or itching sensation on the scalp under the electrodes. In tDCS, a constant low direct current polarises tissue and the direction of current flow is either anodal or cathodal [31]. In studies on the effect of tDCS on the motor cortex by Nitsche et al. in 2000 and 2005, it was shown that anodal tDCS enhances excitability by increasing the spontaneous firing rate, whereas cathodal stimulation hyperpolarises the neuron and thus reduces its excitability [32, 33]. Intensity of tDCS also has an effect on the brain tissue being stimulated as Purpura and McMurty found in their study in 1965 when they demonstrated that pyramidal cells require higher intensity stimulation to activate them than non-pyramidal cells [30]. The long-term and short-term effects of tDCS appear to result from different mechanisms. Liebetanz et al. showed in their 2002 study that carbamazepine (sodium channel blocker) eliminated the effects of anodal tDCS [34] and Nitsche et al. also reported a similar finding with carbamazepine and flunarizine (calcium channel blocker) [35]. Nitsche et al. also found that using NMDA receptor antagonists prevented the longer-term effects of tDCS indicating that while current effects of tDCS depend on membrane polarisation, after effects may be NMDA receptor dependant [35]. A study by Reis et al. showed that tDCS for 5 days led to motor effects that lasted 3 months after the stimulation [36].

**Table 1:** Comparison of rTMS and tDCS

	RTMS	TDCS
Equipment	Pulse generator, stimulation coils	Current generator, electrodes, sponge soaked in saline
Costs	High	Low
Safety aspects/side effects	Risk of fainting and seizures (low) [41]	Skin irritation under electrode, phosphine, nausea, headache, dizziness [42]
Physiological effects	Magnetic field generates action potential in neuron	Direct current increases neurone spontaneous firing rate
Ease of delivery	Relatively difficult requires trained coil holder, large bulky equipment	Relatively easy to apply, equipment is portable

Fregni et al. investigated whether cathodal tDCS to the unaffected hemisphere of stroke patients would improve motor performance and compared this to the effects of anodal stimulation of the affected hemisphere and sham stimulation. They found that cathodal stimulation of the unaffected hemisphere and anodal stimulation of the affected hemisphere both led to improved outcomes in terms of motor recovery. Sham stimulation on the other hand did not have the same effect [37].

The aforementioned studies focussed on motor impairment after stroke but the same effects of tDCS can be applied to patients suffering with dysphagia. Studies involving tDCS were first performed in healthy subjects to establish the ideal stimulation strength to be used in a therapeutic study. Jefferson et al. in 2009 recruited 17 healthy subjects to undergo different strengths and durations of tDCS over several days (anodal 10 min 1 mA, cathodal 10 min 1 mA, anodal 10 min 1.5 mA, cathodal 10 min 1.5 mA, anodal 20 min 1 mA, cathodal 20 min 1 mA, sham). Levels of current needed to induce changes were higher than that need in the hand. It was also found that cortical excitability in the stimulated hemisphere increased after anodal tDCS and decreased after cathodal stimulation [38]. There was no evidence of transcollosal spread which conflicts with the earlier findings of Linderberg et al. in the hand motor cortex.

A study by Kumar et al. in 2011 investigated the effects of tDCS on dysphagia in acute phase stroke patients. 14 patients were randomised to receive either anodal tDCS to the unaffected hemisphere or sham stimulation over 5 consecutive days. Patients who received anodal tDCS had a 2.60 point improvement in DOSS, whereas the sham stimulation group only had an improvement of 1.25 points (*p* = 0.019). 86% of patients (6 out of 7) in the tDCS group had a 2-point DOSS improvement compared with only 43% (3 out of 7) in the sham stimulation group (*p* = 0.107) [39].

More recently, Restivo et al. performed tDCS on dysphagic multiple sclerosis (MS) patients. 18 MS patients were randomised to receive 5 Hz pharyngeal electrical stimulation for 10 min (6 patients), anodal tDCS 2 mA (6 patients), or sham tDCS (6 patients) over the pharyngeal motor cortex for 20 min, for 5 days consecutively. Assessment of patients was with videofluoroscopy, electrophysiology studies and clinical examination, and primary outcomes were variations in the penetration/aspiration scale (PAS) and in the Dysphagia Severity Scale. The most significant improvements were in patients receiving either “real” anodal tDCS and pharyngeal stimulation suggesting that tDCS over the swallowing motor cortex could potentially benefit dysphagic patients with MS [40].

### Systematic Reviews and Meta-Analysis

In the last 5 years, there has been an increase in the number of studies looking at non-invasive brain stimulation in the treatment of neurogenic dysphagia. As a consequence, at least two reviews have been recently published. A systematic review and meta-analysis of non-invasive brain stimulation (NIBS) published in 2015 by Yang et al. pooled the data of many of the aforementioned studies. 6 randomised control trials met the inclusion criteria for this meta-analysis of which 3 were studies using rTMS and 3 were studies using tDCS [43]. All 6 trials compared the intervention with sham stimulation. These trials included patients with dysphagia following cerebrovascular disorders with a total of 59 intervention groups and 55 placebo groups. Outcomes were measured using DOSS, Functional Dysphagia Scale and Videofluoroscopic Dysphagia Scale. All six of the RCTs included in this review demonstrated that either tDCS or rTMS had a positive effect of the severity of dysphagia. The meta-analysis of the studies showed that the immediate dysphagia improvements reported in patients receiving NIBS were statistically significant compared with sham stimulation. The effect of NIBS after 1 and 2 months after the intervention showed a more pronounced and statistically significant improvement compared with sham stimulation. However, when analysing the specific intervention, it was found that only the rTMS intervention resulted in a statistically significant improvement compared with sham stimulation. The tDCS group did not have a statistically significant improvement compared with the sham stimulation group [43].

Another systematic review and meta-analysis of the effect of NIBS on post-stroke dysphagia by Pisegna et al. published in 2016 included 8 RCTs. They concluded that there was a small but significant effect of NIBS on post-stroke dysphagia severity (pooled effect size = 0.55; 95% CI 0.17, 0.93; *p* = 0.004). Three studies included in the meta-analysis had a small negative effect size (Michou et al. 2014; Yang et al. 2012; Kim et al. 2011), whereas the other 5 studies all had positive effect sizes and 2 were statistically significant. The meta-analysis showed that the three tDCS studies had a non-significant effect size (0.52, *p* = 0.12), whereas the 5 studies utilising rTMS showed a larger and significant effect size (0.56, *p* = 0.03) [44].

### Contralesional Versus Ipsilesional Stimulation

Several of the rTMS and tDCS studies mentioned about have used different sites of stimulation to affect dysphagia. As swallowing musculature is represented in both cerebral hemispheres, [11, 45] stimulating either hemisphere could theoretically result in improvements in dysphagia. Yang et al’s meta-analysis showed that while intervention effects were beneficial only in the contralesional stimulation group, the mean standard difference for the ipsilesional stimulation group was greater than that of the contralesional stimulation group (1.05 vs. 0.90). Confidence intervals overlapped between the two groups of stimulation and differences were not statistically significant. From this, Yang et al. concluded that it is not yet possible to determine which stimulation is more effective [43].

By comparison, Pisegna et al’s meta-analysis also explored the difference between contralesional and ipsilesional stimulation. They found that studies stimulating the affected hemisphere had a (non-significant) combined effect size of 0.46 (95% CI −0.18, 1.11; *p* = 0.16), whereas studies stimulating the unaffected hemisphere had a significant combined effect size of 0.65 (95% CI 0.14, 1.16; *p* = 0.01). Hence there may be a stronger argument for focussing NIBS on the unaffected hemisphere in stroke patients at least. There remains uncertainty about the utilisation of NIBS in other conditions such as Parkinson’s disease and motor neuron disease.

### Future Directions

Non-invasive brain stimulation in the form of rTMS and tDCS has come a long way from Valdez and Albert’s early animal experiments through the physiology studies in healthy humans by Hamdy and others, and now to trials in dysphagic stroke patients. It has helped to improve our understanding of the mechanisms underlying motor recovery following brain injury, and both types of stimulation have had positive outcomes in trials treating dysphagia. There remain a lot of unanswered questions regarding the physiological mechanisms of NIBS and the nature of excitatory and inhibitory stimulation, which will require more extensive research in this exciting new field. Further work assessing different stimulation sites, doses and effects on different types of patients will need to be carried out before NIBS truly becomes a viable clinical treatment for dysphagia. However, the early signs are very promising and given the pace of advancement in this field of research, it is not implausible that accessing the full potential of NIBS is close to being realised.
